# Supplementary figures and images for: Characterisation of the interaction of neuropilin-1 with heparin and a heparan sulfate mimetic library of heparin-derived sugars
Source: PeerJ. 2014 Jun 26;2:e461. doi: 10.7717/peerj.461 (PMC4089425; doi:10.7717/peerj.461)

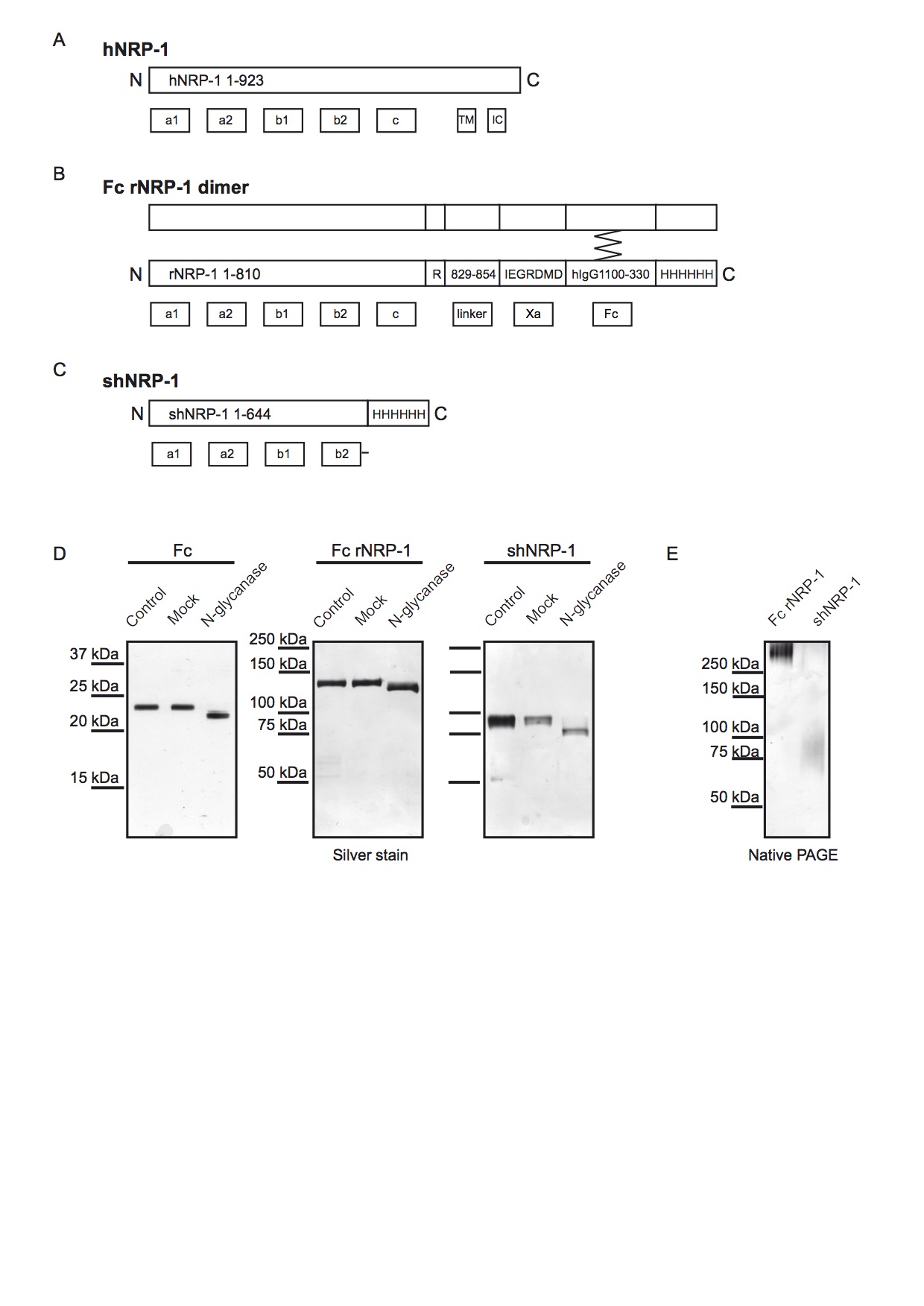

Supplement: Figure S1 — (A) Human full-length NRP-1, where amino acid sequence 1-923 covers the extracellular domains (a–c), transmembrane (TM) and intracellular regions (IC). (B) The Fc linked rat NRP-1, in which two identical molecules are joined by a disulfide bridge within the Fc region. The construct contains all the extracellular domains, which is followed by a small deletion (811-828 amino acids), replaced with arginine residues, subsequent linker region originating from the original rat NRP-1 sequence, a factor Xa cleavage site (IEGRDMD), IgG1 and a His tag. (C) Soluble variant of human NRP-1, where amino acids 1-644 cover the extracellular domains, a1, a2, b1, b2, but not the c domain, and a hexhistidine (6xHis) tag. (D) Silver staining result of N-glycanase digest, performed as described in the experimental section of the reference Fc region, Fc rNRP-1 and shNRP-1, where control, mock digest and the digest results with indicated marker sizes are shown. (E) Silver staining of native PAGE resolution of 100 ng of both recombinant proteins (marker sizes are indicated). [file peerj-02-461-s001.jpg]

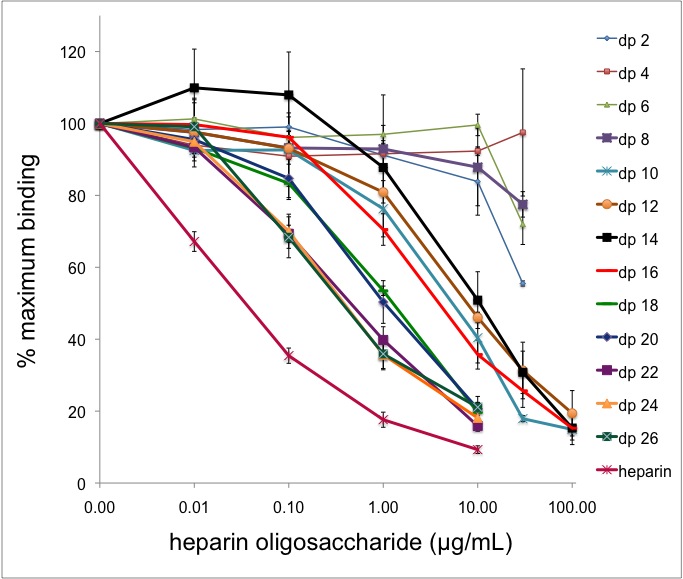

Supplement: Figure S2 — Competition for Fc-NRP-1 binding to the heparin derivatised surface was measured, as described in the experimental section. The relative binding values at each concentration of added sugar competitor were adjusted to 100% of Fc rNRP-1 binding alone to the immobilised heparin (measured at between 10–40 arc s). The experiment was performed three times independently (mean ± SD). [file peerj-02-461-s002.jpg]

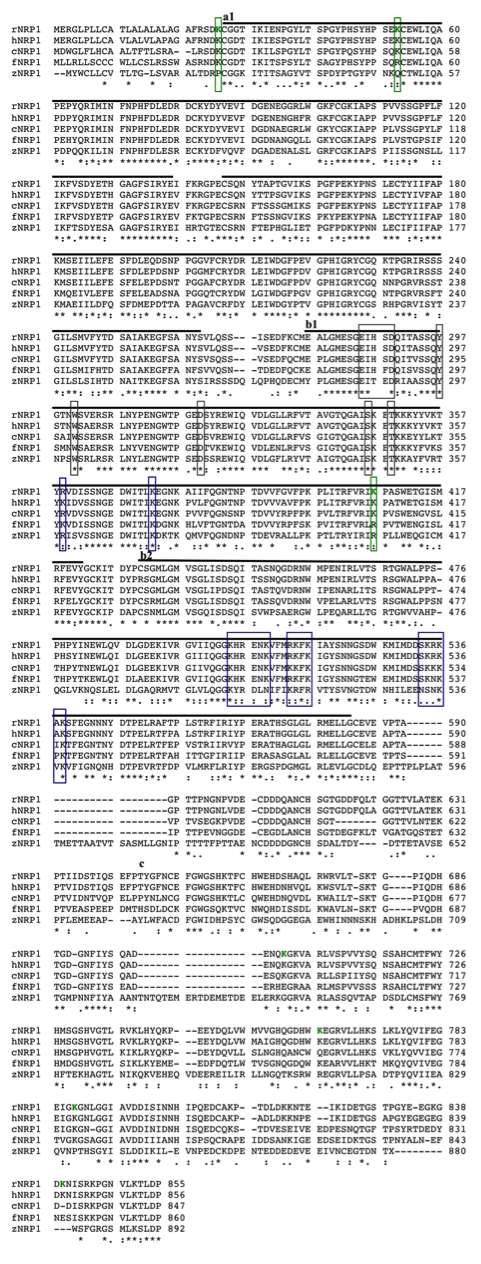

Supplement: Figure S3 — Alignment was performed with Clustal W from the initiator M and the signal sequence through to the end of L2 using sequences retrieved from Uniprot: rNRP1, rat NRP-1, accession number Q9QWJ9; hNRP1, human NRP-1, O14786; cNRP-1, chicken NRP-1, P79795; fNRP1, Xenopus laevis NRP-1; zNRP-1, zebrafish NRP-1, Q8QFX6. Residues identified in (Vander Kooi et al., 2007) as heparin binding are boxed in blue (this construct terminates at amino acid 584), lysines identified in the present work are in green and boxed in green, VEGF binding site (Geretti et al., 2007) is boxed in dark grey. Domains are overlined. [file peerj-02-461-s003.png]

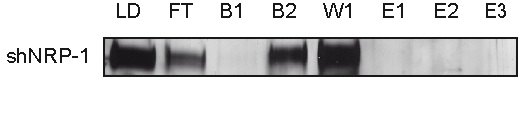

Supplement: Figure S4 — (A) The panel presents the steps of analysis of shNRP-1. Lane (LD) corresponds to the sample (4 µg shNRP-1) applied to the heparin column, lane (FT) is the flow through or unbound material after applying the sample three times to the column, lanes (B1 and B2) are the two acetylation steps, lane (W1) is the post acetylation wash of the column, elution (E1) is the material eluted from the column with 2 M NaCl, lane (E2) is the same material after biotinylation (E2), and (E3) is the biotinylated material after concentration and buffer exchange. Five% (v/v) of each sampl, was analysed on SDS-PAGE and silver stained. [file peerj-02-461-s004.jpg]
