# Supplementary material for: Characterisation of the interaction of neuropilin-1 with heparin and a heparan sulfate mimetic library of heparin-derived sugars
Source: PeerJ. 2014 Jun 26;2:e461. doi: 10.7717/peerj.461 (PMC4089425; doi:10.7717/peerj.461)
Supplement: Figure S5 [file peerj-02-461-s005.pdf]

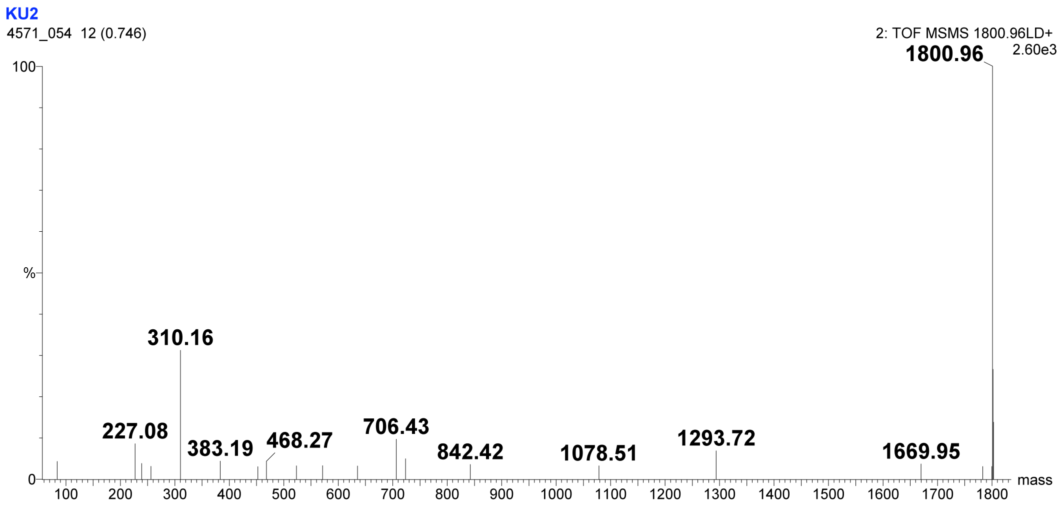

1800.96	*2.60E+03*	*VRIK(Biotin)PASWETGISM*
**Ion fragment mass (m/z)**	**Intensity**	**Assignment (error - ppm)**
84.07	1.11E+02	K(-128)
227.08	2.23E+02	Biotin(0)
239.15	9.89E+01	b_2-NH_3(-1.1)
256.17	8.07E+01	b_2(-27) / PAS(159) / SW-H_2O(242)
310.16	8.10E+02	K(Biotin)(0)
383.19	1.13E+02	ETGI-H_2O(-6.6)
452.24	7.84E+01	K(Biotin)P(16)
468.27	1.11E+02	IK(Biotin)(13)
523.28	8.38E+01	K(Biotin)PA(20)
571.26	8.44E+01	PASWE(16)
635.36	8.28E+01	
706.43	2.51E+02	b_4-NH3(33) / IK(Biotin)PAS-NH_3(100)
723.48	1.28E+02	b_4(64) / IK(Biotin)PAS(130)
842.42	9.22E+01	PASWETGI(19)
1078.51	8.40E+01	y_{10}(21)
1293.72	1.78E+02	b_9(33)
1669.95	9.57E+01	b_{13}+H_2O(46)
1783.01	7.92E+01	MH-H_2O(60)
1799.83	7.92E+01	
1800.96	2.60E+03	MH(26)

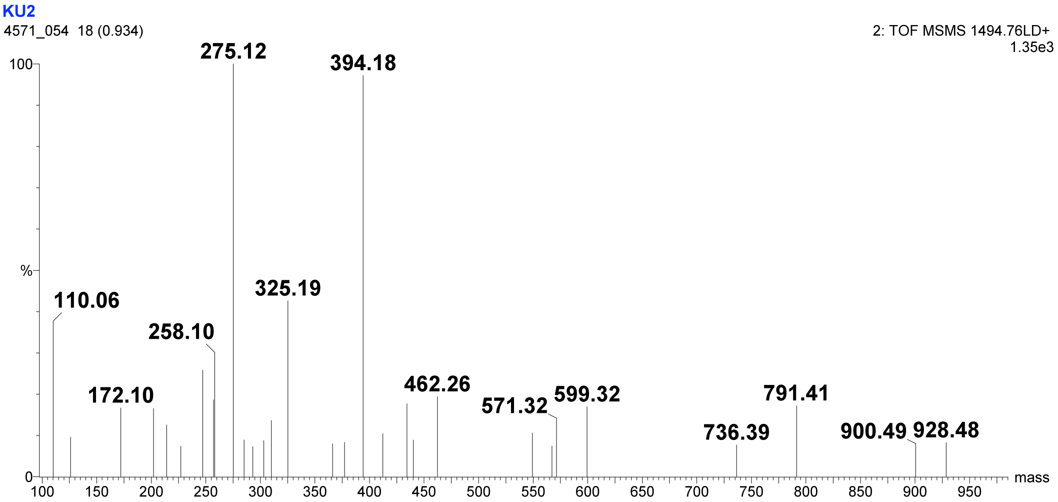

1494.76.	*1.35E+03*	*AGAFR(Acetyl)SDK(Biotin)C(Carbamidomethyl)GGT*
**Ion fragment mass (m/z)**	**Intensity**	**Assignment (error - ppm)**
110.06	5.10E+02	
126.08	1.29E+02	K(-90)
172.1	2.25E+02	a_3(-47)
202.1	2.24E+02	
214.1	1.69E+02	
227.09	9.94E+01	Biotin(44)
247.13	3.49E+02	C(Carbamidomethyl)GG-28(178)
257.11	2.52E+02	
258.1	4.06E+02	
275.12	1.35E+03	C(Carbamidomethyl)GG(142)
285.11	1.21E+02	
293.14	9.84E+01	
303.12	1.18E+02	
310.16	1.84E+02	K(Biotin) (0)
325.19	5.77E+02	
366.18	1.08E+02	
377.16	1.13E+02	
394.18	1.32E+03	y_4(104)
412.22	1.41E+02	
434.26	2.40E+02	
440.19	1.20E+02	
462.26	2.63E+02	
549.26	1.43E+02	
567.27	1.01E+02	
571.32	1.91E+02	
599.32	2.29E+02	
736.39	1.04E+02	
791.41	2.33E+02	
900.49	1.08E+02	
928.48	1.12E+02	

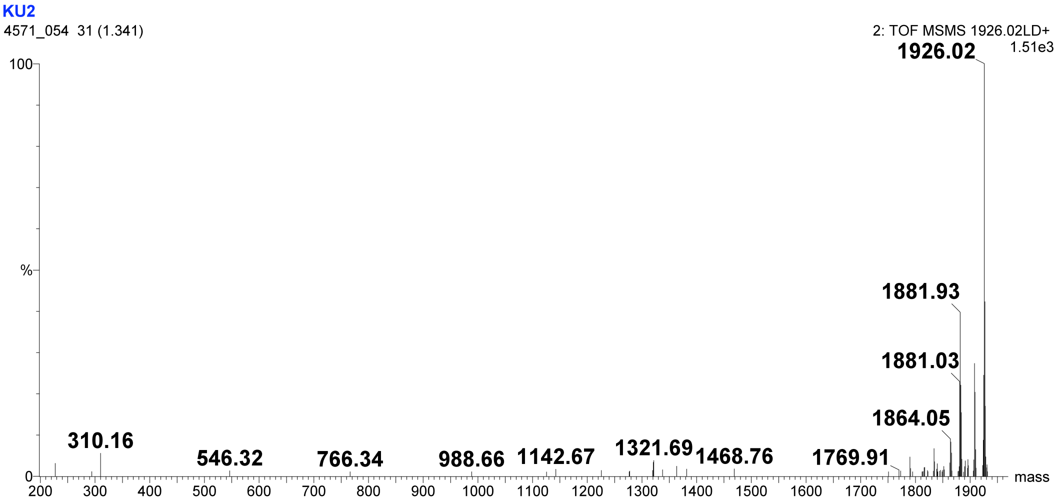

1926.02	*1.51E+03*	*DK(Biotin)NISRKPGNVLKTL(Ammonium)*
**Ion fragment mass (m/z)**	**Intensity**	**Assignment (error - ppm)**
227.08	4.68E+01	Biotin(0)
294.15	1.68E+01	
310.16	8.42E+01	K(Biotin)(0)
546.32	1.99E+01	
766.34	1.62E+01	b_5-H2O(-20)
988.66	1.64E+01	
1125.65	1.56E+01	y_{10}-NH3(-54)
1142.67	2.53E+01	y_{10}(-58)
1225.68	2.08E+01	
1276.69	1.62E+01	
1319.63	2.09E+01	b_{10}-NH3(-17)
1337.74	2.35E+01	
1363.73	3.65E+01	
1381.71	2.62E+01	
1468.76	2.67E+01	
1750.95	1.60E+01	
1769.91	2.24E+01	
1789.98	7.06E+01	
1812.03	1.58E+01	
1832.87	1.88E+01	
1862.96	4.89E+01	
1877.99	1.71E+01	
1905.84	1.91E+01	
1923	3.95E+01	

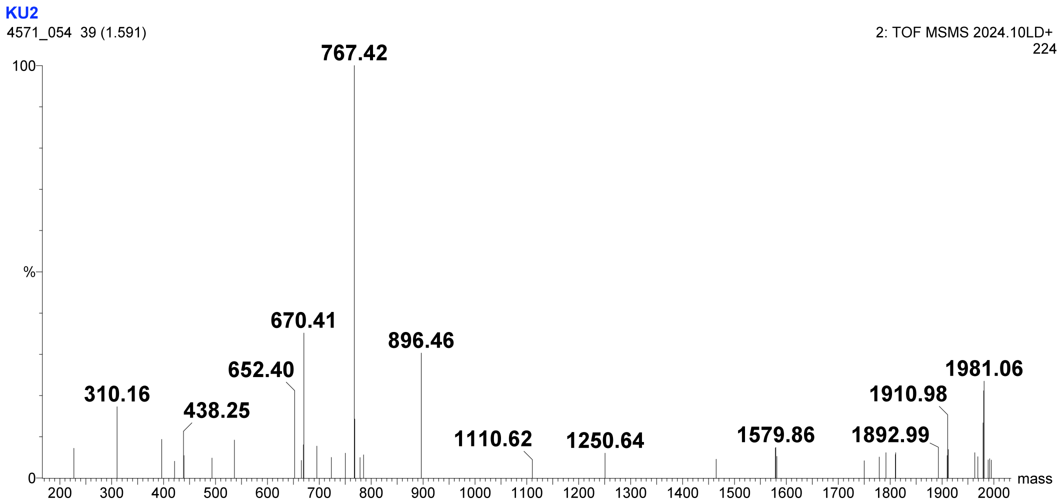

2024.10	*224*	*TLPPSRDELTK(Biotin)NQVSL*
**Ion fragment mass (m/z)**	**Intensity**	**Assignment (error - ppm)**
227.08	1.60E+01	Biotin(0)
310.16	3.86E+01	K(Biotin)(0)
396.23	2.09E+01	
421.22	9.04E+00	PPSR-NH_3(1.4)
438.25	2.52E+01	PPSR(9.3) / TK(Biotin)-H_2O(75) / PSRD-H_2O(92)
493.25	1.08E+01	
536.27	2.05E+01	PPSRD-NH_3(44)
652.4	4.72E+01	b_6(34)
665.29	9.50E+00	PPSRDE-NH_3(1.6) / LTK(Biotin)N-H2O(-81)
669.43	1.80E+01	
695.44	1.72E+01	
723.38	1.11E+01	
750.37	1.34E+01	b_7-NH_3(-11)
767.42	2.24E+02	PPSRDEL-28(20) / b_7(20) / LPPSRDE-28(20)
778.42	1.10E+01	LPPSRDE-NH_3(60) / PPSRDEL-NH3(60)
785.41	1.25E+01	DELTK(Biotin)-28(30)
896.46	6.77E+01	y_6-H_2O(-6.5) / PPSRDELT(14) / b_8(14)
1110.62	9.71E+00	y_8-H_2O(20) / b_{10}(37)
1250.64	1.34E+01	PPSRDELTK(Biotin)(16) / PSRDELTK(Biotin)N-NH_3(45)
1464.77	1.01E+01	b_{11}(13) / PPSRDELTK(Biotin)NQ-28(30)
1578.81	1.62E+01	b_{12}(9.8)
1749.9	9.31E+00	
1778.93	1.13E+01	
1791.97	1.37E+01	LPPSRDELTK(Biotin)NQVS(36) / y_{14}-H_2O(36)
1810	1.27E+01	y_{14}(46)
1892.99	1.65E+01	b_{15}(19)
1910.06	1.21E+01	
1963.1	1.37E+01	
1968.98	1.15E+01	
1979.15	2.99E+01	
1989	9.74E+00	
1995.08	9.66E+00	

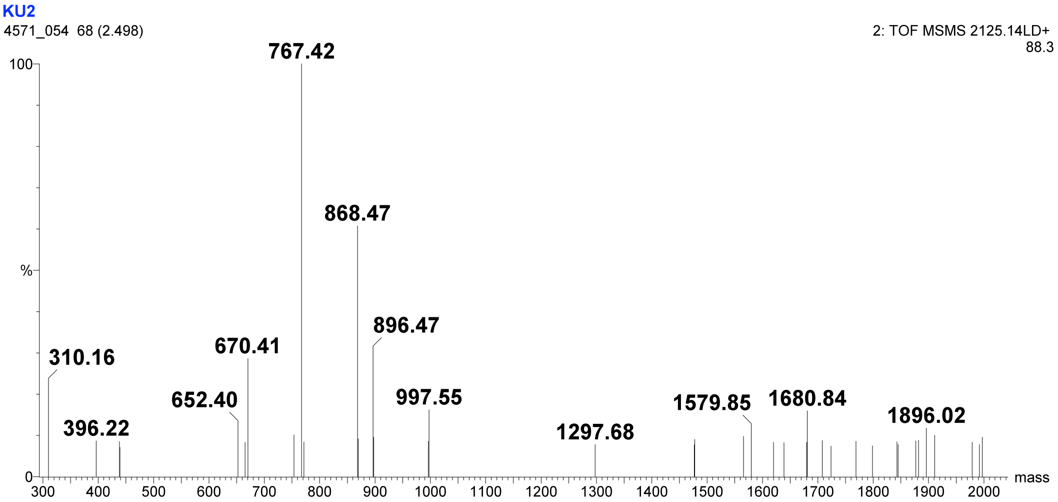

2125.14	*88.30*	*VVVDVSHEDPEVK(Biotin)FNW*
**Ion fragment mass (m/z)**	**Intensity**	**Assignment (error - ppm)**
310.16	2.10E+01	K(Biotin)(0)
396.22	7.65E+00	
438.25	7.50E+00	
652.4	1.19E+01	PEVK(Biotin)-28(79)
665.29	7.35E+00	VSHEDP(1.6) / DVSHED-H_2O(56)
670.41	2.53E+01	
753.44	8.94E+00	
767.42	8.83E+01	DPEVK(Biotin)-28(58)
771.49	7.40E+00	
868.47	5.37E+01	
896.47	2.79E+01	EDPEVK(Biotin)-28(58)
996.48	7.55E+00	
1297.68	6.86E+00	
1476.73	6.81E+00	
1565.76	8.62E+00	
1579.85	1.13E+01	
1619.84	7.36E+00	
1638.8	7.27E+00	
1679.94	7.36E+00	VVDVSHEDPEVK(Biotin)F-28(78)
1708.02	7.71E+00	VVDVSHEDPEVK(Biotin)F(126)
1723.92	6.52E+00	
1768.98	7.58E+00	
1798.82	6.59E+00	
1842.84	7.47E+00	
1876.88	7.62E+00	
1881.84	7.78E+00	
1896.02	1.04E+01	
1911.03	8.83E+00	
1978.9	7.34E+00	
1991.94	6.89E+00	
1996.97	8.44E+00	

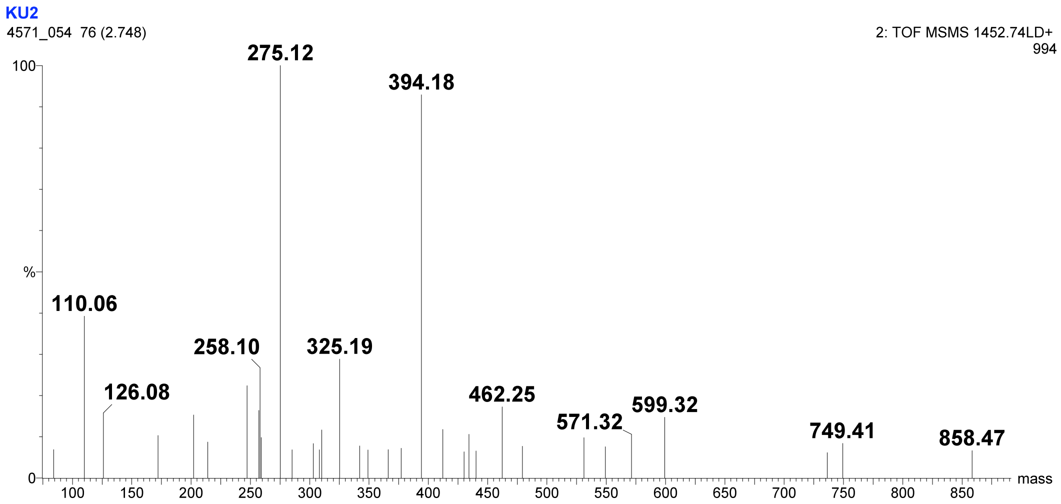

1452.74	*994*	*AGAFRSDK(Biotin)C(Carbamidomethyl)GGT*
**Ion fragment mass (m/z)**	**Intensity**	**Assignment (error - ppm)**
84.07	6.80E+01	K(-128)
110.06	3.90E+02	
126.08	1.56E+02	K(-90)
172.1	1.02E+02	a_3(-47)
202.1	1.52E+02	
214.1	8.64E+01	
247.13	2.22E+02	C(Carbamidomethyl)GG-28(178)
257.11	1.63E+02	
275.12	9.94E+02	C(Carbamidomethyl)GG(142)
285.11	6.76E+01	
303.12	8.27E+01	
308.17	6.77E+01	
310.16	1.16E+02	K(Biotin)(0)
325.19	2.86E+02	
342.21	7.69E+01	RSD-NH_3(202)
349.15	6.73E+01	
366.18	6.80E+01	
377.15	7.16E+01	
394.18	9.24E+02	y_4(104)
412.21	1.17E+02	
430.2	6.30E+01	
434.26	1.05E+02	AFRS-28(21)
440.19	6.48E+01	
462.25	1.71E+02	AFRS(8.8)
479.29	7.63E+01	
531.27	9.69E+01	
549.29	7.51E+01	AFRSD-28(22)
571.32	1.05E+02	
599.32	1.46E+02	
736.39	6.08E+01	
749.41	8.32E+01	
858.47	6.56E+01	

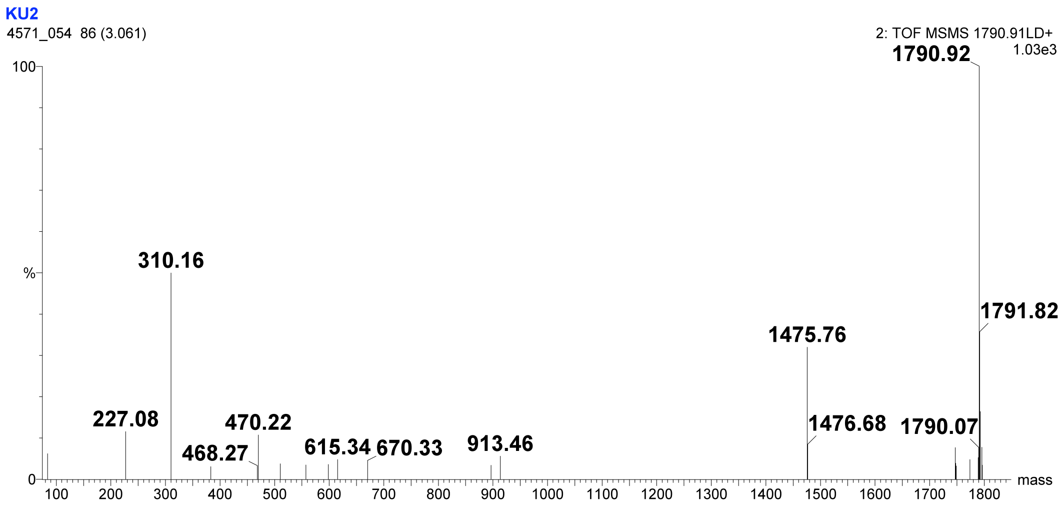

1790.92	*1.03E+03*	*TVDK(Biotin)SRWQQGNVF*
**Ion fragment mass (m/z)**	**Intensity**	**Assignment (error - ppm)**
84.07	6.36E+01	K(-128) Q(-128)
227.08	1.19E+02	Biotin(0)
310.16	5.17E+02	K(Biotin)(0)
383.17	3.11E+01	
468.27	3.36E+01	
510.23	3.86E+01	QQGNV-NH_3(-1.3)
557.26	3.54E+01	DK(Biotin)S(38)
598.33	3.69E+01	K(Biotin)SR(28)
615.34	4.90E+01	
670.33	4.64E+01	b_4(11)
896.43	3.45E+01	b_6-NH_3(0.60)
913.46	5.76E+01	b_6(4.4)
1475.76	3.30E+02	y_{10}(26)
1746.86	7.88E+01	
1773.88	4.91E+01	MH-NH_3(24)
1788.84	5.40E+01	

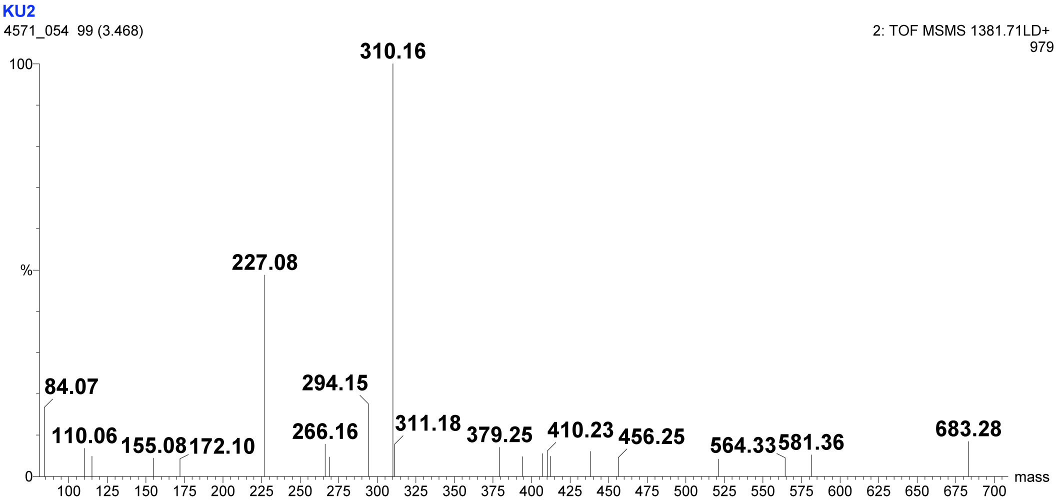

1381.71	*979*	*NGK(Biotin)EYKCKVS*
**Ion fragment mass (m/z)**	**Intensity**	**Assignment (error - ppm)**
84.07	1.63E+02	K(-128)
110.06	6.61E+01	
115.08	4.71E+01	
155.08	4.28E+01	b_2-NH_3(225)
172.1	4.09E+01	b_2(165)
227.08	4.78E+02	Biotin(0)
266.16	7.58E+01	
269.16	4.50E+01	
294.15	1.71E+02	
310.16	9.80E+02	K(Biotin)(0)
379.25	6.82E+01	
394.23	4.65E+01	
407.25	5.35E+01	
438.22	5.87E+01	
456.25	4.39E+01	K(Biotin)E-28(49)
521.33	4.06E+01	
564.33	4.34E+01	y_5(22)
581.36	5.02E+01	
683.28	8.24E+01	

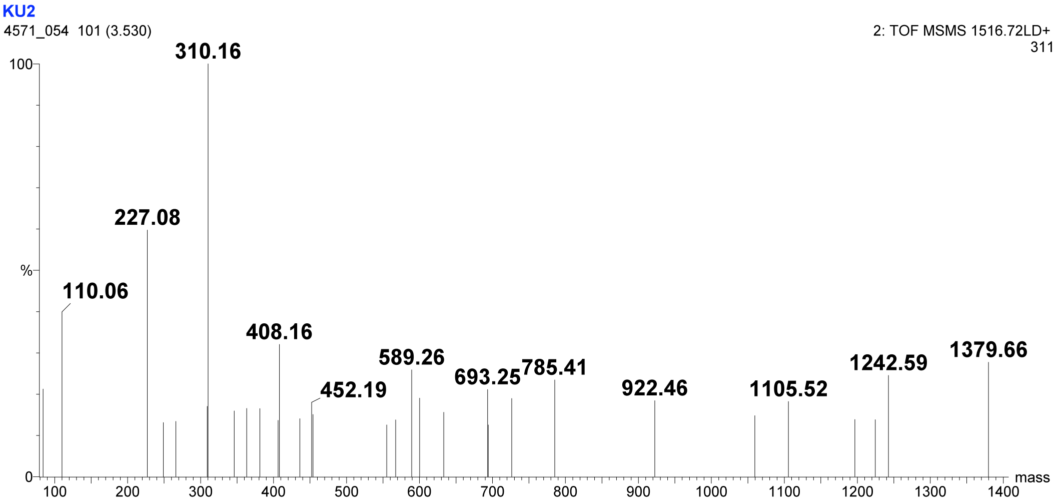

1516.72	*311*	*QVIFEGEIGK(Biotin)GN*
**Ion fragment mass (m/z)**	**Intensity**	**Assignment (error - ppm)**
84.07	6.59E+01	Q(-128) / K(-128)
110.06	1.24E+02	
227.08	1.86E+02	Biotin(0)
249.09	4.07E+01	FE-28(-134)
266.12	4.16E+01	
310.16	3.11E+02	K(Biotin)(0)
346.12	4.94E+01	
363.14	5.14E+01	
381.15	5.12E+01	
406.18	4.23E+01	
436.15	4.36E+01	
452.19	5.58E+01	GK(Biotin)G-NH_3(-14)
567.26	4.28E+01	
589.26	8.03E+01	a_5(-126)
600.26	5.91E+01	b_5-NH_3(-71)
633.24	4.85E+01	FEGEIG(-76)
693.25	6.56E+01	EIGK(Biotin)G-H_2O(-128) / GEIGK(Biotin)-H_2O(-128)
726.32	5.89E+01	
785.41	7.28E+01	b_7-H_2O(35)
922.46	5.72E+01	
1059.5	4.59E+01	
1105.52	5.66E+01	
1196.57	4.29E+01	
1224.61	4.29E+01	
1242.59	7.62E+01	
1379.66	8.61E+01	

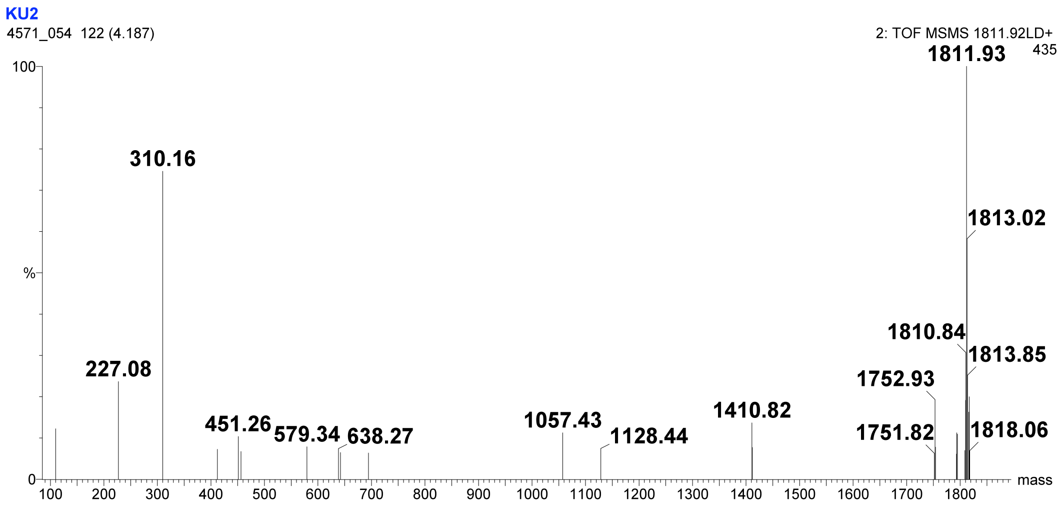

1811.92	*435*	*SQADENQK(Biotin)GK(Acetyl)VARL*
**Ion fragment mass (m/z)**	**Intensity**	**Assignment (error - ppm)**
110.06	5.30E+01	
227.08	1.03E+02	Biotin(0)
310.16	3.24E+02	K(Biotin)(0)
412.21	3.14E+01	K(Biotin)G(21) / ADEN-H_2O(155)
451.26	4.48E+01	
456.15	2.92E+01	
579.34	3.41E+01	
638.27	3.20E+01	
642.24	2.81E+01	
694.37	2.75E+01	
1057.43	4.88E+01	
1128.44	3.21E+01	
1410.82	5.93E+01	y_{10}(48)
1751.82	2.70E+01	
1792.99	2.64E+01	
1808.83	3.02E+01	
1813.02	2.53E+02	

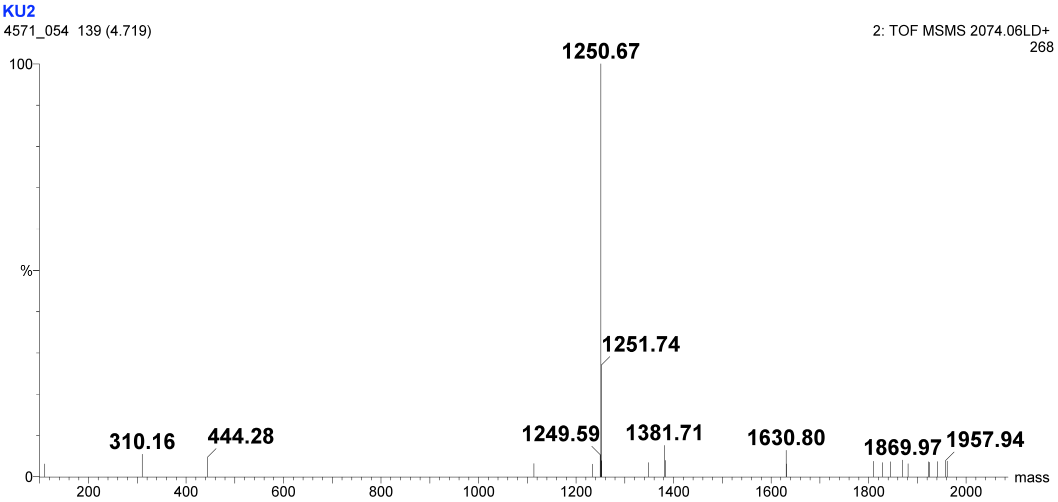

2074.06	*268*	*MVVGHQGDHWK(Biotin)EGRVL*
**Ion fragment mass (m/z)**	**Intensity**	**Assignment (error - ppm)**
110.06	8.21E+00	H(-102)
310.16	1.45E+01	K(Biotin)(0)
444.28	1.26E+01	y_4(-29)
1113.68	8.41E+00	y_7(83)
1233.62	8.10E+00	y_8-NH_3(0.22)
1249.59	1.41E+01	
1348.77	9.12E+00	y_9-NH_3(91)
1381.71	2.02E+01	
1630.8	1.71E+01	b_{12}(46)
1809.94	9.99E+00	
1828.9	9.11E+00	
1845	9.63E+00	
1869.97	1.08E+01	
1880.93	8.29E+00	
1923.14	9.60E+00	
1940.98	9.75E+00	
1957.94	1.03E+01	
1961.06	1.01E+01	b_{15}+H_2O(68)

KU2

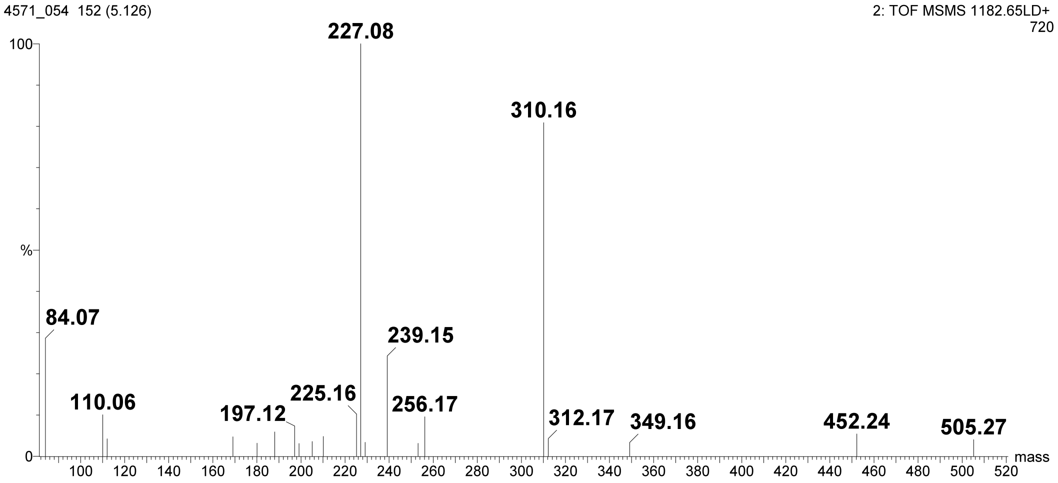

1182.65	*720*	*VRIK(Biotin)PASW*
**Ion fragment mass (m/z)**	**Intensity**	**Assignment (error - ppm)**
84.07	2.06E+02	K(-128)
110.06	7.22E+01	
169.12	3.38E+01	PA(135)
180.09	2.26E+01	
188.06	4.23E+01	
197.12	5.23E+01	
205.1	2.56E+01	y_1(14)
210.15	3.43E+01	
225.16	7.30E+01	
227.08	7.20E+02	Biotin(0)
239.15	1.74E+02	b_2-NH_3(-1.1)
253.16	2.22E+01	RI-NH_3(-23)
256.17	6.88E+01	b_2(-27) / PAS(159)
310.16	5.82E+02	K(Biotin)(0)
349.16	2.34E+01	
452.24	3.88E+01	K(Biotin)P(16)
505.27	2.89E+01	

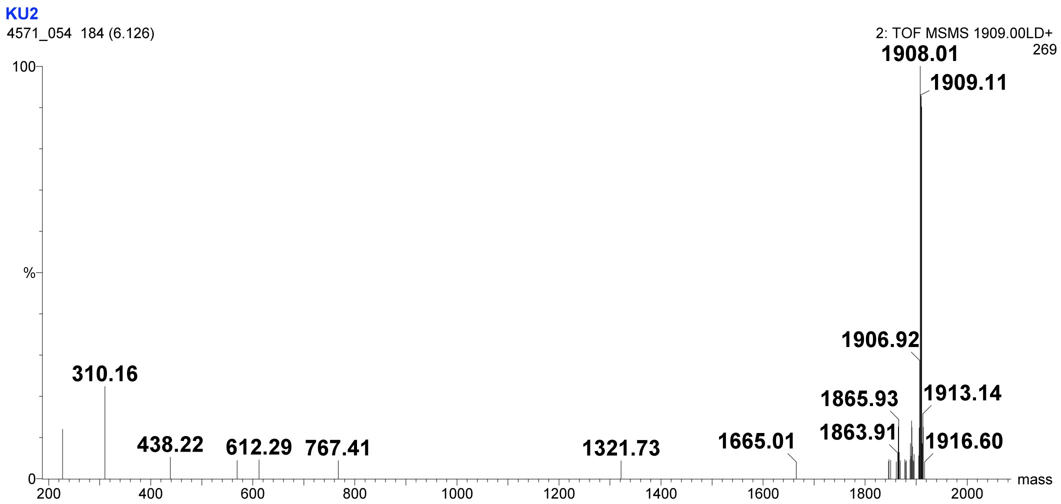

1909.00	*269*	*DK(Biotin)NISRKPGNVLKTL*
**Ion fragment mass (m/z)**	**Intensity**	**Assignment (error - ppm)**
227.08	3.23E+01	Biotin(0)
310.16	6.01E+01	K(Biotin)(0)
438.22	1.40E+01	NVLK-NH_3(-117)
569.27	1.19E+01	
612.29	1.23E+01	SRKPGN-28(-110)
767.41	1.17E+01	b_5-NH_3(92)
1321.73	1.17E+01	
1665.01	1.08E+01	
1845.97	1.13E+01	
1860.93	1.09E+01	
1877.95	1.25E+01	
1880.95	1.18E+01	
1887.98	1.25E+01	
1904.95	1.49E+01	
1911.01	2.42E+02	
1916.6	1.08E+01	

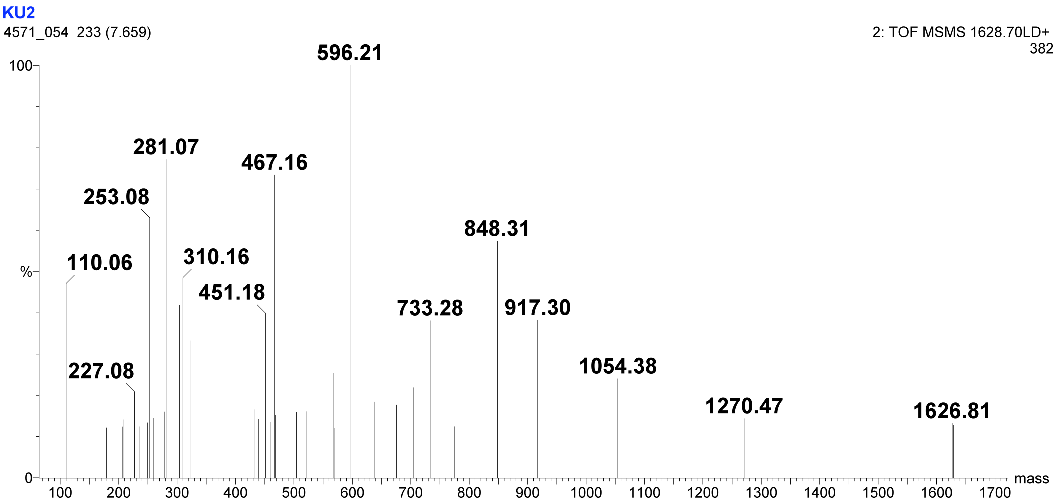

1628.70	*382*	*HSYHPSEK(Biotin)CEW*
**Ion fragment mass (m/z)**	**Intensity**	**Assignment (error - ppm)**
110.06	1.80E+02	H(-102)
179.09	4.61E+01	a_2-H_2O(-15)
207.08	4.71E+01	b_2-H_2O(-37) / HP-28(-213)
227.08	7.94E+01	Biotin(0)
235.09	4.71E+01	HP(-123)
249.1	5.08E+01	
253.08	2.41E+02	
260.11	5.52E+01	
278.12	6.09E+01	
281.07	2.95E+02	
304.1	1.60E+02	HPS-H_2O(-133)
310.16	1.85E+02	K(Biotin)(0)
322.12	1.27E+02	HPS(-96)
433.15	6.31E+01	HPSE-H_2O(-76)
439.16	5.39E+01	
451.18	1.52E+02	HPSE(-30)
459.18	5.16E+01	
467.16	2.80E+02	EK(Biotin)-NH_3(-77) / YHPS-H_2O(-94) / SYHP-H_2O(-94)
504.2	6.07E+01	
522.23	6.12E+01	
568.23	9.65E+01	
596.21	3.82E+02	YHPSE-H_2O(-61)
637.26	7.01E+01	
675.3	6.73E+01	
705.27	8.34E+01	
733.28	1.45E+02	
774.31	4.73E+01	y_4-NH_3(19)
848.31	2.19E+02	
917.3	1.46E+02	
1054.38	9.17E+01	YHPSEK(Biotin)C-NH_3(-30)
1270.47	5.47E+01	SYHPSEK(Biotin)CE-NH_3(-13)
1626.81	5.02E+01	

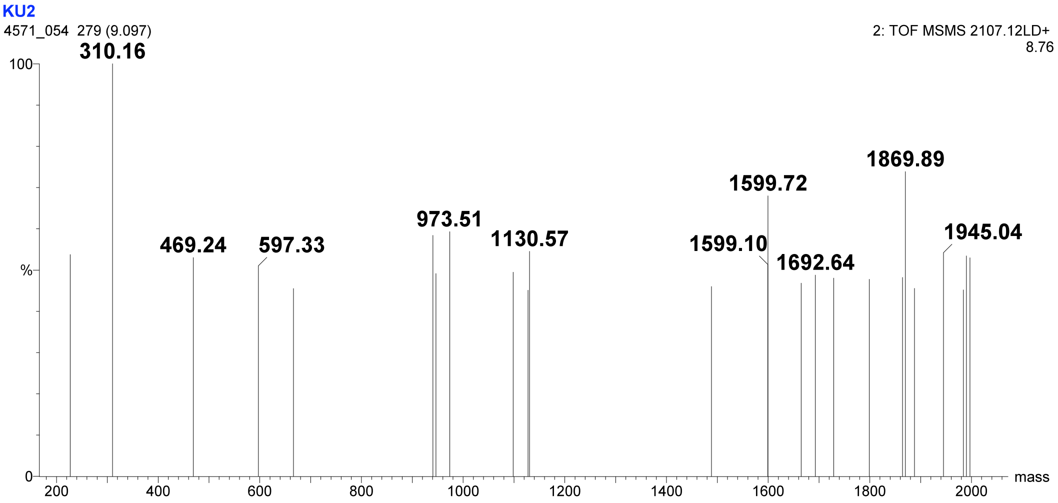

2107.12	*8.76*	*VVVDVSHEDPEVK(Biotin)FNW -H_2O*
**Ion fragment mass (m/z)**	**Intensity**	**Assignment (error - ppm)**
469.24	4.64E+00	
597.33	4.46E+00	
666.29	3.98E+00	
940.45	5.11E+00	
946.45	4.30E+00	
973.51	5.19E+00	
1098.54	4.33E+00	
1127.55	3.95E+00	y_7-H_2O(14)
1130.57	4.77E+00	SHEDPEVK(Biotin)-H_2O(68)
1488.52	4.03E+00	
1599.1	4.49E+00	
1664.88	4.10E+00	
1692.64	4.27E+00	
1729	4.21E+00	
1799.09	4.18E+00	
1864.16	4.22E+00	
1869.89	6.47E+00	
1887.92	3.99E+00	
1945.04	4.74E+00	
1983.99	3.96E+00	
1990.07	4.68E+00	y_{15}-H_2O(77)
1997.1	4.64E+00	

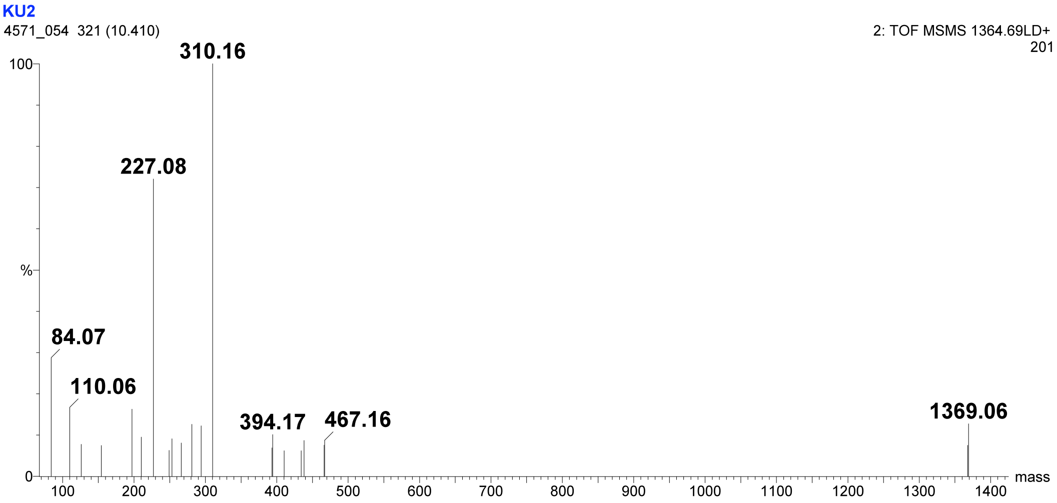

1364.69	*201*	*NGK(Biotin)EYKCKVS -NH_3*
**Ion fragment mass (m/z)**	**Intensity**	**Assignment (error - ppm)**
84.07	5.77E+01	K(-128)
110.06	3.35E+01	
126.08	1.55E+01	K(-90)
154.08	1.50E+01	
197.12	3.27E+01	
210.16	1.90E+01	
227.08	1.45E+02	Biotin(0)
249.09	1.25E+01	
253.08	1.82E+01	
266.16	1.62E+01	
281.07	2.53E+01	
294.16	2.46E+01	
310.16	2.01E+02	K(Biotin)(0)
393.2	1.38E+01	EYK-28(-34)
410.26	1.24E+01	
434.15	1.24E+01	
438.23	1.74E+01	
466.22	1.51E+01	K(Biotin)E-H_2O(17)
